# High levels of halogenated natural products in large pelagic fish from the Western Indian Ocean

**DOI:** 10.1007/s11356-021-14738-0

**Published:** 2021-06-15

**Authors:** Qiong Wu, Catherine Munschy, Yann Aminot, Nathalie Bodin, Walter Vetter

**Affiliations:** 1 Institute of Food Chemistry (170b), University of Hohenheim, Garbenstraße 28, D-70599 Stuttgart, Germany; 2 Laboratory of Biogeochemistry of Organic Contaminants, IFREMER (French Research Institute for Exploitation of the Sea), Rue de l’île d’Yeu, BP 21105, 44311 Nantes Cedex 3, France; 3 Fishing Port, SFA (Seychelles Fishing Authority), Victoria, Mahé, Seychelles; 4 Fishing Port, IRD (French Research Institute for Sustainable Development), Victoria, Mahé, Seychelles; 5 Present Address: SOS (Sustainable Ocean Seychelles), BeauBelle, Mahé, Seychelles

**Keywords:** Seychelles, Marine predator, Swordfish, Tropical tuna, Polyhalogenated compound, DDT, PCB, HNP

## Abstract

Concentrations, profiles and muscle-liver distribution of halogenated natural products (HNPs) and anthropogenic persistent organic pollutants (POPs) were investigated in five large pelagic fish species and one smaller planktivore fish species from the Western Indian Ocean. Analysis of swordfish muscle from the Seychelles revealed the predominance of HNPs, with the highest concentrations found for 2′-methoxy-2,3′,4,5′- tetraBDE (2′-MeO-BDE 68 or BC-2), 6-methoxy-2,2′,4,4′- tetraBDE (6-MeO-BDE 47 or BC-3) and 2,3,3′,4,4′,5,5′-heptachloro-1′-methyl-1,2′-bipyrrole (Q1), along with varied contributions of further HNPs. The mean concentration of ∑HNPs (330 ng/g lw) was one or two orders of magnitude higher than ∑DDTs (60 ng/g lw) and ∑PCBs (6.8 ng/g lw). HNPs (BC-2, BC-3 and Q1) were also predominant in individual samples of three tropical tuna species from the Seychelles and from other regions of the Western Indian Ocean (Mozambique Channel, off Somalia and Chagos Archipelago). Non-targeted gas chromatography coupled with electron capture negative ion mass spectrometry operated in the selected ion monitoring mode (GC/ECNI-MS-SIM) analysis of one swordfish sample indicated low abundance of rarely reported HNPs (three hexachloro-1′-methyl-1,2′-bipyrrole (Cl_6_-MBP) isomers and pentabromo-1,1′-dimethyl-2,2′-bipyrroles (Br_5_-DBP)) but no further abundant unscreened polyhalogenated compounds.

**Supplementary Information:**

The online version contains supplementary material available at 10.1007/s11356-021-14738-0.

## Introduction

Anthropogenic persistent organic pollutants (POPs) have been frequently detected in marine environmental and biological samples (Wenning and Martello 2014; Qiu et al. 2020). High trophic level marine species in particular, such as mammals and predatory fish, may be burdened with high levels of POPs due to bioaccumulation through the food chain (Hoondert et al. 2020; Hop et al. 2002). Chronic exposure to POPs can pose serious health risks to these organisms (Sonne et al. 2020). Therefore, these POPs have been banned (e.g. PCBs and several chlorinated pesticides) or restricted (e.g. DDT and perfluorooctane sulfonate (PFOS)) for production and used by the Stockholm Convention because of their bioaccumulative, persistent and toxic properties (Kim et al. 2014). In addition, several other polyhalogenated compounds with structures and physicochemical properties similar to POPs have been repeatedly detected in environmental samples. About ten compounds or compound classes of these lipophilic polyhalogenated compounds were found to be of natural origin (Bidleman et al. 2019; Teuten et al. 2005; Vetter 2006, 2012). The natural production of certain HNPs (such as BC-2 and BC-3) has been verified by their isolation from whale blubber followed by radiocarbon measurements (Teuten et al. 2005). These halogenated natural products (HNPs) belong to a huge variety of over 5000 HNPs which are mainly produced by certain lower marine organisms such as sponges, worms, bacteria and algae (Gribble 2012). Although toxicity and potential risk to human health are largely unknown (Gribble 2012), these few lipophilic HNPs are of particular concern because they can be found in the diet of humans. In 2016, HNPs had been recognized as emerging contaminants by the Arctic Monitoring and Assessment Programme (AMAP) (Wilson 2018).

**Fig. 1:**
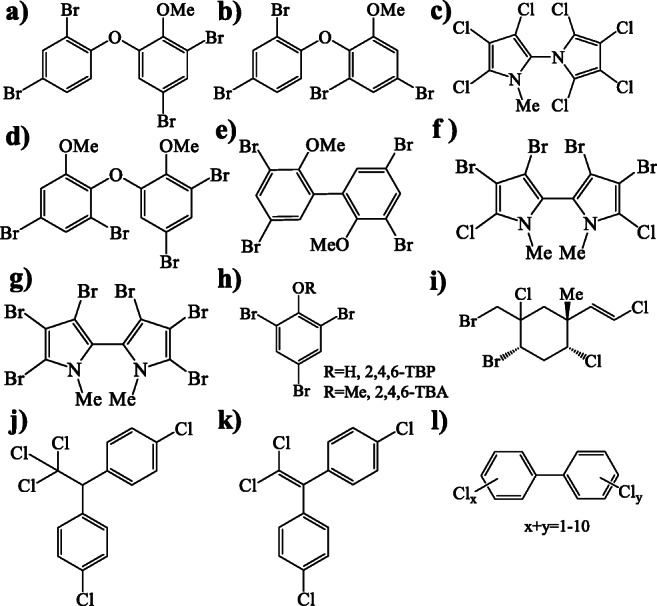
Chemical structures of **a** 2′-methoxy-2,3′,4,5′- tetraBDE (2′-MeO-BDE 68 or BC-2), **b** 6-methoxy-2,2′,4,4′- tetraBDE (6-MeO-BDE 47 or BC-3), **c** 2,3,3′,4,4′,5,5′-heptachloro-1′-methyl-1,2′-bipyrrole (Q1), **d** 3,5-dibromo-2-(3′,5′-dibromo,2′-methoxy)phenoxyanisole (BC-11), **e** 2,2′-dimethoxy-3,3′,5,5′-tetrabromobiphenyl (2,2′-diMeO-BB80 or BC-1), **f** 1,1′-dimethyl-3,3′,4,4′-tetrabromo-5,5′-dichloro-2,2′-bipyrrole (BC-10), **g** 1,1′-dimethyl-3,3′,4,4′,5,5′-hexabromo-2,2′-bipyrrole (Br_6_-DBP), **h** 2,4,6-tribromophenol/tribromoanisole (2,4,6-TBP/-TBA), **i** (1*R*,2*S*,4*R*,5*R*,1′*E*)-2-bromo-1-bromomethyl-1,4-dichloro-5-(2′-chloroethenyl)-5-methylcyclohexane (MHC-1), **j** 1,1,1-trichloro-2,2-bis(4-chlorophenyl)ethane (*p,p*′-DDT), **k** 1,1-dichloro-2,2-bis(4-chlorophenyl)ethane (*p,p*′-DDE) and **l** polychlorinated biphenyls (PCBs)

Although HNPs have been detected in many marine regions worldwide (Alonso et al. 2017; Goto et al. 2020; Löfstrand et al. 2010; Malmvärn et al. 2005; Shaul et al. 2015; Teuten and Reddy 2007; Wu et al. 2019), one commonly known hotspot of HNPs is the Great Barrier Reef in Australia (Vetter et al. 2001, 2002, 2009). Several HNPs such as 2,3,3′,4,4′,5,5′-heptachloro-1′-methyl-1,2′-bipyrrole (Q1) or the tetrabrominated methoxylated diphenyl ethers (2′-MeO-BDE 68 or BC-2, and 6-MeO-BDE 47 or BC-3) were detected at ppm-levels in marine mammals inhabiting the Great Barrier Reef (Fig. 1a–i), thus confirming their bioaccumulative nature (Vetter et al. 2001, 2002). The Great Barrier Reef (coordinates 18° 11′ S, 147° 27′ E) is characterized by a rich flora, appreciable climate with warm ocean water and the presence of ~900 tropical islands (Johnson and Marshall 2007). Arguably, these conditions are favourable for the natural production of various HNPs.

One marine region with similar geographic and climate characteristics is the Seychelles archipelago (coordinates 4° 35′ S, 55° 40′ E). Located approximately 1500 km off the coast of Africa in the Indian Ocean, the Seychelles archipelago is located in the same temperature zone (tropical) as the Great Barrier Reef. Also, the Seychelles consist of 115 islands spread over a large economic exclusive zone (EEZ: 1.37 million km^2^). Despite the favourable conditions for their natural production, the occurrence of HNPs in Seychelles and more largely in the Western Indian Ocean has not been explored so far.

In this study, we thus aimed to get first insights into the occurrence of HNPs in the Seychelles pelagic ecosystem. For this purpose, we first investigated several classes of HNPs (Fig. 1a–i) and POPs (i.e. polychlorinated biphenyl (PCBs, Fig. 1l) and dichlorodiphenyltrichloroethane (DDT) and its metabolites (Fig. 1j, k)) in the muscle and liver of Seychelles swordfish. Since HNP levels may vary strongly within short distances (Hauler et al. 2014; Malmvärn et al. 2005; Vetter et al. 2009) and across different species (Wu et al. 2019, 2020b), we then compared HNP and POP concentrations in swordfish with those in individual samples of other fish species from the same habitat. Samples analysed included three tropical tuna species (yellowfin tuna, bigeye tuna and skipjack tuna) and one newborn silky shark. In addition to these large pelagic fish species, we also screened one potential forage fish, the small pelagic Indian mackerel, on polyhalogenated compounds. Further, tropical tunas from the Chagos Archipelago, off Somalia coasts and Mozambique Channel (~2000 km from Seychelles) were analysed to verify the widespread occurrence of HNPs in marine biota of the Western Indian Ocean. Since several HNPs were initially described in samples from the Great Barrier Reef, we also thoroughly analysed one carefully selected Seychelles swordfish sample by means of a non-target gas chromatography coupled with electron capture negative ion mass spectrometry operated in the selected ion monitoring mode (GC/ECNI-MS-SIM) approach (Hauler and Vetter 2015) in order to cover the full array of polyhalogenated compounds and possibly hitherto unknown or non-monitored contaminants.

## Materials and methods

### Sample collection

Four large pelagic fish, i.e. swordfish *Xiphias gladius* (n=10), yellowfin tuna *Thunnus albacares* (n=1), bigeye tuna *Thunnus obesus* (n=1) and skipjack tuna *Katsuwonus pelamis* (n=1), were caught in the Seychelles EEZ between January 2013 and January 2014. A newborn silky shark *Carcharhinus falciformis* (n=1) and one smaller pelagic fish Indian mackerel *Rastrelliger kanagurta* (n=1) were caught in August and March 2018, respectively, in the Seychelles EEZ. In addition, yellowfin tuna (n=1), bigeye tuna (n=1) and skipjack tuna (n=1) were collected off the coasts of Somalia, in the Mozambique Channel and an area located offshore between Seychelles and the Chagos archipelago (hereafter referred to as Chagos) in March–July 2013 (Fig. S1, Table S1). The swordfish and Indian mackerel were caught by a commercial long liner, and the tropical tunas and silky shark were sampled by a commercial purse seiner. Individual fish was measured and sexed directly on board of the fishing vessel: the low jaw fork length was taken for the swordfish, the fork length for the three tropical tunas and the Indian mackerel and the standard length for the silky shark. Based on the very small body length (69 cm, Table S1), the silky shark was a newborn (length at birth ~72 cm compared to adult males and females measuring 210–220 cm and >225 cm, respectively (Branstetter 1987)).

### Chemicals

Sources and quality of chemicals and standards used for sample clean-up (trace analysis quality) were reported in details by Munschy et al. (2020). Additional chemicals used for HNP analysis were *iso*-octane (>99.0%) from Riedel-de Haen (Steinheim, Germany). The internal standard 2,3-dibromopropyl-2,4,6-tribromophenyl ether (DPTE) for quantification was synthesized in our laboratory (von der Recke and Vetter 2007). Standards of ICES-7 PCBs (PCB 28, PCB 52, PCB 101, PCB 118, PCB 138, PCB 153, PCB 180) were ordered from Dr. Ehrenstorfer (Augsburg, Germany). Origins and quality of HNP standards were reported in details by Wu et al. (2019). Mixed standards were prepared from stock solutions stored at −20°C in accurately weighed vials. After condition to ambient temperature, vials were weighed and only used when the weight matched the previously noted weight at less than 2% since its generation. Further instrumental controls were based on peak abundance relative to the internal standards.

### Sample storage and clean-up

Fish were stored frozen on board until landing. After landing, a subsample (~5 cm^3^) was carefully taken from the front dorsal white muscle (sampled under the dorsal spine on the left side) from each fish along with a liver sample from the Seychelles swordfish (n=4). All samples were frozen (amber glassware, −20°C) followed by freeze-drying at SFA Seychelles Fishing Authority Research Laboratory. Freeze-dried samples were sent to LBCO at Ifremer, Nantes, France, for organic contaminant and total lipid content analysis. The fish samples were processed as described by Munschy et al. (2020). Briefly, each freeze-dried and ground sample was extracted with dichloromethane using accelerated solvent extraction (ASE 300, Dionex, USA) and then successively purified using gel permeation chromatography, a silica and alumina column and a two-dimensional HPLC system with two columns coupled in series (Munschy et al. 2020). Aliquots of each purified sample extract were manually transported to the University of Hohenheim for analysis of HNPs and POPs. Here, extract volumes were set to ~200 μL and then spiked with 20 μL of DPTE solution (2 ng/μL) used as internal standard for quantification and last constant volume with *iso*-octane to 270 μL.

### Instrumental analysis

HNPs and POPs were quantified by gas chromatography combined with electron capture negative ion mass spectrometry (GC/ECNI-MS) performed with an Agilent 7890/5975C system (Waldbronn, Germany) according to Bendig et al. (2013). Briefly, the sample solution (1 μL) was injected into a programmed temperature vaporizer injector (CIS-4, Gerstel, Mülheim, Germany) operated in splitless mode. The temperature of transfer line, ion source and quadrupole were set at 300°C, 150°C and 150°C, respectively. The reagent gas methane 5.5 (Air Liquide, Bopfingen, Germany) was introduced with a flow rate of 40 mL/min. An Optima 5 MS (30 m, 0.25 mm internal diameter, 0.25 μm film thickness, Macherey-Nagel, Düren, Germany) was installed in the GC oven. During injection, the GC oven temperature was kept at 50°C. After 1 min, it was raised at 10°C/min to 300°C, which was held for 14 min. Polyhalogenated compounds were quantified in selected ion monitoring (SIM) mode according to Wu et al. (2019). Initial screening on PBDEs indicated that BDE 47 was the most abundant PBDE congener in all samples, with very low mean concentrations of 0.5 and 0.4 ng/g lw for BDE 47 in tuna and swordfish, respectively. Therefore, PBDE concentrations will not be discussed in this study. The non-target GC/ECNI-MS-SIM analysis was carried out according to Vetter et al. (2017), which covered the range *m/z* 248-704 in twelve runs (names NT1 to NT12) and five time windows.

### Quality assurance and quality control (QA/QC)

Samples were prepared in the French laboratory (LBCO at Ifremer) as described above and shipped to the German laboratory (University of Hohenheim) for analysis of HNPs and POPs. Procedural blanks were free of HNPs and the internal standard DPTE, but some of them contained traces of PCBs and DDTs, which were subtracted when analysing samples. The recovery of ^13^C-labelled compounds of PCBs and DDTs in samples were within 76–88%. Limit of detection (LOD) and limit of quantification (LOQ) were determined for all target analytes using the corresponding standards by the three-fold and ten-fold signal-to-noise (S/N) ratio, respectively (Table S2). To exclude concentration changes due to the shipment of sample solutions, ΣPCB concentrations were determined by GC/ECNI-MS in the German lab and compared with those initially determined in France via gas chromatography coupled with high-resolution mass spectrometry in electron ionization mode (GC/EI-HRMS) using a 50 m × 0.22 mm internal diameter × 0.2 μm film thickness HT-8 column (Munschy et al. 2020). Concentrations obtained by GC/EI-HRMS (recovery corrected) were generally slightly higher compared to GC/ECNI-MS (not recovery corrected). Namely, median PCB concentrations determined by GC/EI-HRMS and GC/ECNI-MS were 5.4 and 3.5 ng/g lw, respectively. The two concentrations showed a significant linear correlation (r^2^=0.892, *p*<0.01), indicating that the method used in this study (GC/ECNI-MS) was reliable, and the difference between the concentrations of PCBs obtained by the two methods may be due to the different quantitation methods (for instance, PCB and DDT levels as determined by GC/EI-HRMS were recovery corrected but not the current ones determined by GC/ECNI-MS). Since recovery correction was not possible for HNPs, we will also present POP levels without corrections. Statistical software SPSS 16.0 (SPSS, Chicago, IL, USA) was used for regression analysis.

## Results and discussion

### Levels of HNPs and anthropogenic POPs in swordfish from the Seychelles waters

**Fig. 2:**
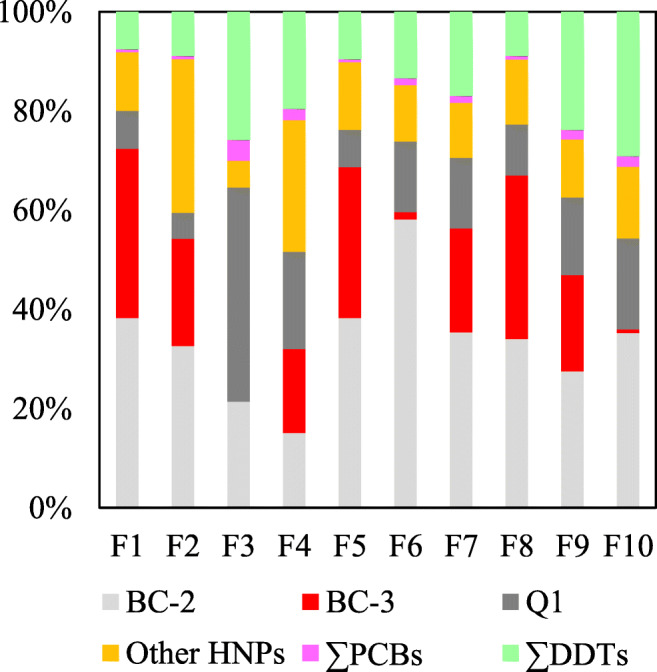
Contributions (%) of different HNPs and anthropogenic POPs to the total amount of polyhalogenated compounds in swordfish muscle (n=10) from the Seychelles waters in the Western Indian Ocean

**Fig. 3:**
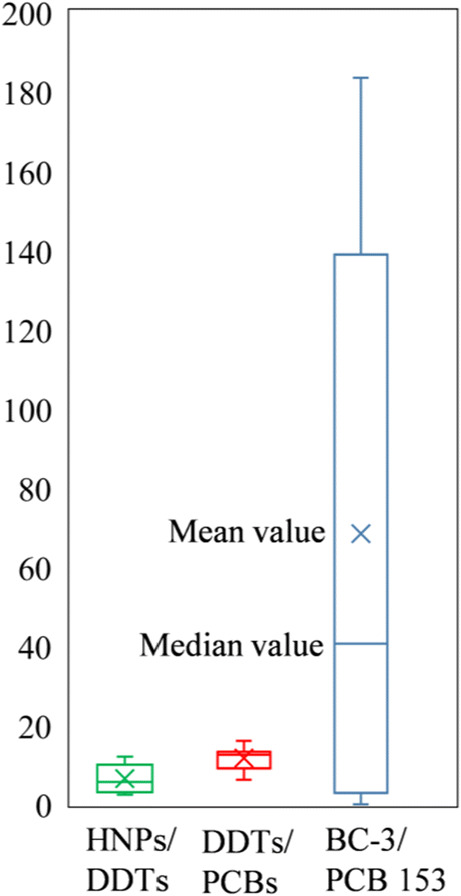
Ratios of HNPs/DDTs, DDTs/PCBs and BC-3/PCB 153 in swordfish muscle (n=10) from the Seychelles waters in the Western Indian Ocean

Initial GC/ECNI-MS screening of the swordfish muscle samples (n=10) in the full scan mode indicated a pronounced predominance of HNPs over anthropogenic POPs. Namely, ΣHNPs accounted for >70% (up to 92%) of the total concentrations of target halogenated contaminants (Fig. 2). Typically, concentration ranges of individual HNPs varied by about one order of magnitude (Table 1). Seven HNPs or HNP classes were detected with predominance of BC-2, BC-3 and Q1 (Figs. 1a–c and 2). Except for 3,5-dibromo-2-(3′,5′-dibromo,2′-methoxy)phenoxyanisole (BC-11) (Fig. 1d), the detection rates of all polyhalogenated compounds were high (≥ 90%, Table 1). Concentrations decreased in the order ΣHNPs > ΣDDTs > ΣPCBs from 45–590 (mean value: 330 ng/g lw) to 19–190 (mean value: 60 ng/g lw) to 1.4–31 ng/g lw (mean value: 6.8 ng/g lw), respectively, i.e. a range of about one order of magnitude, in each case (Table 1). More constant concentration ratios of ΣDDTs/ΣPCBs (R_DDT/PCB_: 12±2.8, 6.2–16) compared to ΣHNPs/ΣDDTs (R_HNP/DDT_: 6.5±3.7, 2.4–12) in swordfish muscles indicated that the distribution of major POPs was similar but different from HNPs (Fig. 3). In agreement with that, ΣDDTs showed a significant positive correlation with ΣPCBs (Fig. S2a, r^2^ = 0.9747, *p* < 0.01), but not with ΣHNPs (Fig. S2b, r^2^ = 0.2815, *p* > 0.05). Therefore, the occurrence of anthropogenic POPs was more predictable compared to HNPs. In agreement with that, the concentration range of the HNP BC-3 was more variable and spanned over three orders of magnitude (Table 1). This led to highly varying BC-3/PCB 153 ratios (Fig. 3). Also, the swordfish sample F3 showed the highest concentration of Q1, PCBs and DDTs but not of BC-2 and particularly BC-3 which was <LOQ (Table 1). Despite these individual variations, mean concentrations of ΣHNPs, ΣDDTs and ΣPCBs agreed well with the corresponding median values (Table 1). Therefore, further discussion will be based on mean values.

**Table 1:** Concentrations of HNPs and anthropogenic POPs (ng/g lw) in the muscle of swordfish (n=10) from the Seychelles waters in the Western Indian Ocean

Code	Sampling date	Gender ^a^/length (cm)	BC-2	BC-3	Q1	BC-11	BC-1	BC-10	Br_6_-DBP	2,4,6-TBA	MHC-1	∑HNPs	∑DDTs	∑PCBs	R_HNP/DDT_	R_DDT/PCB_
F1	Jan.2013	F/163	240	220	49	5.6	12	22	27	4.7	4.0	590	48	3.7	12	13
F2	Dec.2013	M/187	200	130	32	140	12	12	24	1.6	1.3	560	55	4.1	10	13
F3	Dec.2013	F/193	160	<LOQ	320	<LOQ	17	19	<LOQ	1.6	2.4	520	190	31	2.7	6.2
F4	Apr.2013	M/204	74	83	96	94	18	<LOQ	13	2.3	2.6	380	96	11	4.0	8.7
F5	Jun.2013	F/180	130	110	26	12	6.0	11	15	1.5	1.4	310	33	2.1	9.4	16
F6	Nov.2014	F/203	200	5.0	48	<LOQ	8.5	15	9.2	4.9	0.8	290	45	4.9	6.4	9.2
F7	Nov.2014	M/160	120	68	46	<LOQ	7.1	17	8.7	2.0	1.2	270	55	4.7	4.8	12
F8	Sep.2013	F/150	96	93	29	<LOQ	5.6	9.9	17	2.6	1.7	260	25	2.2	10	11
F9	Nov.2014	M/121	37	26	21	<LOQ	3.6	6.2	3.0	1.8	1.2	100	32	2.5	3.1	13
F10	Nov.2014	M/151	23	0.5	12	<LOQ	2.1	4.0	2.3	0.9	0.1	45	19	1.4	2.4	14
Range in males		121–204	23–200	0.5–130	12–96	<LOQ–140	2.1–18	<LOQ–17	2.3–13	0.9–2.3	0.1–2.6	45–560	19–96	1.4–11	2.4–10	8.7–14
Range in females		150–203	96–240	<LOQ–220	26–320	<LOQ–12	5.6–17	9.9–22	<LOQ–27	1.5–4.9	0.8–4.0	260–590	25–190	2.2–31	2.7–12	6.2–16
Range		121–204	23–240	<LOQ-220	12–320	<LOQ–140	2.1–18	<LOQ–22	<LOQ–27	0.9–4.9	0.1–4.0	45–590	19–190	1.4–31	2.4–12	6.2–16
Median		172	120	76	39	<LOQ	7.8	12	11	1.9	1.4	300	47	3.9	5.6	12
Mean		171	130	73	68	25	9.2	12	12	2.4	1.7	330	60	6.8	6.5	12

2,4,6-TBP was <LOD in all samples

^a^*F* female, *M* male

^b^R_HNP/DDT_=∑HNPs/∑DDTs, R_DDT/PCB_=∑DDTs/∑PCBs

**Fig. 4:**
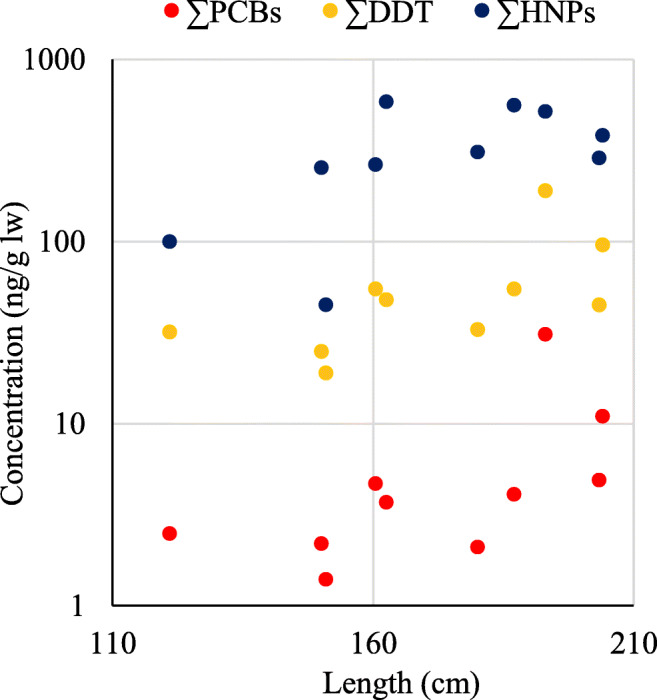
Correlation between fish length (cm) and concentrations of halogenated contaminants (∑HNPs, ∑DDT and ∑PCBs) (ng/g lipid weight) determined in the swordfish muscle (n=10) from the Seychelles waters in the Western Indian Ocean

Mean and range concentrations of ΣHNPs, ΣPCBs, ΣDDTs as well as R_DDT/PCB_ and R_HNP/DDT_ showed no significant difference (*p* < 0.05) between the female (n=5) and male (n=5) swordfish samples (Table 1). Also, no significant correlation between ΣHNPs (r^2^= 0.33, *p*>0.05), ΣPCBs (r^2^=0.21, *p*>0.05) and ΣDDTs (r^2^=0.27, *p*>0.05) with swordfish length was observed in the present study (Fig. 4).

In addition to muscle, liver samples were available from swordfish samples F1, F3, F5 and F10. Preferential accumulation of HNPs, PCB 153 and *p,p’*-DDE was studied by means of the lipid-based concentration ratio of liver/(liver+muscle) (R_L/L+M_) according to Weijs et al. (2009). Accordingly, R_L/L+M_ > 0.5 indicated preferential accumulation in liver, while R_L/L+M_ < 0.5 indicated higher abundance in muscle (Voorspoels et al. 2003). For all HNPs except Q1, results were inconsistent in the four swordfish livers (Table 2). For instance, R_L/L+M_ values of BC-2 were <0.5 in sample F1 and F5, but they were >0.5 in sample F3 and F10 (Table 2). One reason could be variations in the dietary intake of HNPs (indicative of hotspots) combined with a non-equilibrium partitioning between the two tissues. Especially, in the case of HNPs, it is known that concentrations can strongly vary in low trophic biota (prey) within short distances (Hauler et al. 2014; Barón et al. 2015; Estrella et al. 2018). By contrast, R_L/L+M_ values for PCB 153 and *p,p’*-DDE were ≤0.5 (Table 2). In agreement with that, similar R_L/L+M_ values for ΣPCBs and *p,p´*-DDE (0.31 and 0.30, respectively) could be calculated from literature data in swordfish from both the Southern Ionian Sea (Corsolini et al. 2005) and the Southern Tyrrhenian Sea (< 0.5 for *p,p'-*DDE) (Corsolini et al. 2008). Interestingly, Q1 also showed R_L/L+M_ values <0.5 (or 0.5 in sample F5). This indicated that the distribution of Q1 within this habitat was more even than the one of other HNPs such as the BCs and rather similar to anthropogenic POPs. In addition, R_L/L+M_ of Q1 was significantly correlated with R_L/L+M_ of PCB 153 (r^2^=0.9637, *p*<0.05) but not with R_L/ L+M_ of *p,p’*-DDE (r^2^=0.1655, *p*>0.05) (Fig. S3a,b). It has been suggested that Q1 could be produced by marine bacteria, but most of other HNPs were produced by algae and sponges (Gribble 2003; Vetter 2006). Therefore, the HNPs produced by marine bacteria could show a different muscle-liver distribution in the present samples.

**Table 2:** Liver/(liver + muscle) lipid-based concentration ratios of HNPs and anthropogenic POPs in swordfish from the Seychelles waters in the Western Indian Ocean

Code	BC-2	BC-3	Q1	BC-11	BC-1	BC-10	Br_6_-DBP	2,4,6-TBA	MHC-1	PCB 153	*p,p’*-DDE
F1	0.31	0.31	0.31	**0.64**	0.31	0.24	0.34	0.36	0.29	0.22	0.27
F3	**0.69**	n.a.	0.22	n.a.	0.43	0.26	n.a.	**0.82**	0.35	0.15	0.37
F5	0.47	0.48	0.50	0.48	n.a.	**0.69**	0.44	**0.58**	**0.53**	0.50	0.46
F10	**0.78**	**0.99**	0.41	n.a.	**0.82**	n.a.	**0.83**	**0.85**	**0.92**	0.33	0.28

*n.a.* not applicable (concentrations <LOQ)

Values highlighted in bold indicate preferential accumulation in liver.

In agreement with this, POP concentrations in Seychelles swordfish were similar to those found in lower trophic level biota from South Africa coast (Wu et al. 2020b). Moreover, PCB 153 (0.5–2.6 ng/g lw) and *p,p´*-DDE (1.8–26 ng/g lw) were the predominant anthropogenic pollutants in most swordfish muscle samples (Munschy et al. 2020) (Table S3). These POP concentrations were much lower than those found in swordfish from the Mediterranean Sea (340 and 880 ng/g lw for PCB 153 and *p,p’*-DDE, respectively) (Stefanelli et al. 2004). This was not surprising since the Mediterranean Sea is a semi-enclosed and densely populated ocean area, and human activities were more likely to pollute the ocean, and the seawater is renewed less frequently than in the open ocean (Western Indian Ocean) (Marsili et al. 2018). In contrast, low levels of PCB 153 (12 ng/g lw) and *p,p’*-DDE (13 ng/g lw) were detected in swordfish from a Brazilian EEZ in the Atlantic Ocean (de Azevedo e Silva et al. 2007).

### Comparison of swordfish contamination with other large pelagic and a small pelagic species from the Seychelles waters

HNPs were additionally quantified in individual muscle samples of four large pelagic species (tropical yellowfin, bigeye and skipjack tuna, respectively, and a newborn silky shark) as well as one sample of Indian mackerel (a small pelagic forage fish and potential prey of the pelagic predators).

**Fig. 5:**
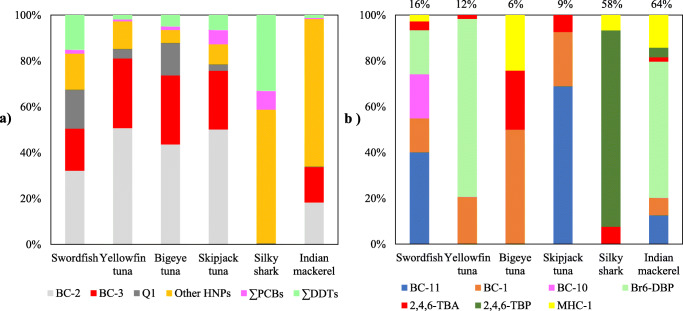
**a** Contributions (%) of different HNPs and anthropogenic POPs to the total amount of polyhalogenated compounds in muscle of swordfish (mean value), yellowfin tuna, bigeye tuna, skipjack tuna, silky shark and Indian mackerel from the Seychelles waters and **b** corresponding detailed contributions of other HNPs

Similar to the swordfish and the three tropical tunas, ∑HNPs accounted for more than 90% of the total target contaminant concentrations in Indian mackerel, and the total HNP concentration (280 ng/g lw) was also within the range of the four large pelagic fish species (110–670 ng/g lw) (Table 3, Fig. 5a). However, the HNP profile of the Indian mackerel differed from the other species (Fig. 5a). Namely, 1,1′-dimethyl-3,3′,4,4′,5,5′-hexabromo-2,2′-bipyrrole (Br_6_-DBP) was the predominant compound in the Indian mackerel sample (~40% of ΣHNPs), and its concentration (110 ng/g lw Br_6_-DBP) was higher than in all swordfish and tropical tuna samples from the Seychelles (≤ 64 ng/g lw Br_6_-DBP) (Table 3). Additionally, concentrations of (1*R*,2*S*,4*R*,5*R*,1′*E*)-2-bromo-1-bromomethyl-1,4-dichloro-5-(2′-chloroethenyl)-5-methylcyclohexane (MHC-1) and 2,4,6-tribromophenol (2,4,6-TBP) in the forage fish were also higher than those in the large pelagic species (Table 3). Since Indian mackerels are planktivores and the predators are opportunistic feeders, different habitats and migration ranges may cause Indian mackerel only to play a minor role in the predators’ diet (Sardenne et al. 2016). In addition, fish could be able to partly metabolize some less persistent HNPs similarly to some POPs. For instance, stable carbon isotope analysis indicated that fish were able to partly metabolize MHC-1 (Rosenfelder and Vetter 2012). Under this premise, HNP levels in marine organisms may be more dependent on their actual abundance in their habitat. As an example, MHC-1 was highly abundant in mussels from Heligoland (German island in the North Sea), because Heligoland is a major habitat for a natural producer of MHC-1, i.e. the red seaweed *Plocamium cartilagineum* (Wu et al. 2020a). By contrast, MHC-1 concentrations in mussels at Hörnumtief (Germany), which is only ~60 km from Heligoland, were only ~1% of those at Heligoland (Hauler et al. 2014). Hence, HNP concentrations in forage fish with smaller migration range would be more affected by HNP hotspots than in large pelagic fish with wider migration range (“dilution effect”). Typically, contaminant evaluations are based on a number of samples from the same region, which are considered representative for a wider area. In the case of HNPs, positions of hotspots are currently very difficult to predict (Hauler et al. 2014; Wu et al. 2020b). Lower concentrations in highly migratory and opportunistic predator species at a given location could then be the result of both a more efficient metabolism of HNPs but also a diluting effect caused by the intake of various diet items (e.g., fish, cephalopod, crustaceans) from different areas. The latter effect could be the explanation for a similar observation made by Weijs et al. (2009). In their study from the Southern North Sea, Weijs et al. (2009) detected lower concentrations of MeO-PBDEs in larger harbour porpoises and harbour seals than in smaller individuals.

**Table 3:** Concentrations of HNPs and anthropogenic POPs (ng/g lw) in the muscle of five large pelagic fish (swordfish, the three tropical tunas and silky shark) and one small pelagic fish (Indian mackerel) from the Seychelles waters in the Western Indian Ocean

	Sampling date	BC-2	BC-3	Q1	BC-11	BC-1	BC-10	Br_6_-DBP	2,4,6-TBA	2,4,6-TBP	MHC-1	∑HNPs	*∑DDTs	*∑PCBs	R_DDT/PCB_
Swordfish	Jan.2013-Nov.2014	130	73	68	25	9.2	12	12	2.4	<LOQ	1.7	330	60	6.8	12
Yellowfin tuna	Feb.2013	350	210	29	<LOD	17	<LOQ	64	1.4	<LOQ	<LOQ	670	13	6.0	2.2
Bigeye tuna	Apr.2013	52	36	17	<LOD	3.3	<LOQ	<LOQ	1.7	<LOQ	1.6	110	5.8	2.0	2.9
Skipjack tuna	Apr.2013	120	60	6.9	14	4.8	<LOQ	<LOQ	1.5	<LOQ	<LOQ	210	15	15	1.0
Silky shark	Aug.2018	<LOQ	<LOQ	0.1	<LOQ	<LOQ	<LOQ	<LOQ	0.8	9.1	0.7	11	6.0	1.5	4.0
Indian mackerel	Mar.2018	52	44	0.8	23	14	<LOQ	109	3.5	7.7	26	280	3.5	1.4	2.5

*Concentrations of individual DDT and PCB congeners were shown in Table S3

Interestingly, while the same HNPs predominant in swordfish were also the most relevant ones in the three tropical tuna species (Fig. 5a), the relevance of other HNPs which contributed between 6 and 16% to the total HNP concentrations was subject to variations (Fig. 5b). Some HNPs like BC-11 or 1,1′-dimethyl-3,3′,4,4′-tetrabromo-5,5′-dichloro-2,2′-bipyrrole (BC-10) were only present in one or two species but not in the other ones. This underlines the particular role of HNPs, which were much higher concentrated than PCBs, if present. Likewise, the Indian mackerel featured a higher number of HNPs than the pelagic predators.

A unique HNP pattern was also detected in muscle tissue of an additional pelagic predator species, the silky shark, from the same region. The total contamination of this newborn individual (<20 ng/g lw) was one order of magnitude lower than in swordfish (400 ng/g lw) and tropical tunas (120–690 ng/g lw) (Table 3). This was mainly due to lower HNP concentrations, which in turn resulted in a higher share of POPs (41% of the total contamination, Fig. 5a). Also, the HNP pattern differed strongly from the other fish species (Fig. 5a, b). Namely, the silky shark did not show measurable concentrations of the otherwise dominating BC-2 and BC-3, and the concentration of Q1 was also very low (Fig. 5a, Table 3). A further peculiarity in the HNP pattern of this silky shark was the high abundance of 2,4,6-TBP which could not be detected in the other large pelagic species (Table 3, Fig. 5b). One reason could be a low accumulation rate of 2,4,6-TBP because of its comparably low log *K*_*OW*_ value of only 3.9 and its phenolic character (Aptula et al. 2002). Hence, halogenated phenolic compounds would be more abundant in serum than in lipids (similarly to hydroxylated PCBs (Letcher et al. 2000)). Instead, tropical tuna and swordfish featured higher concentrations of its metabolite, i.e. 2,4,6-tribromoanisol (2,4,6-TBA) (Nyholm et al. 2009). Little is known about the transformation of 2,4,6-TBP into 2,4,6-TBA. However, two laboratory studies indicated a low biomagnification potential and rapid elimination rate of 2,4,6-TBP in zebrafish (freshwater fish) with half-lives of less than 2 days, while its metabolite (2,4,6-TBA) was persistent and bioaccumulated in the fish (Haldén et al. 2010; Nyholm et al. 2009). In addition, newborn silky sharks may feed on a lower trophic level with a higher abundance of 2,4,6-TBP. For instance, 2,4,6-TBP was also higher concentrated than 2,4,6-TBA in Indian mackerel (Fig. 5b). However, these hypotheses should be further verified with higher number of samples and other low trophic level species in future. Irrespective of the mostly scattered sample number, HNPs were generally more abundant than POPs (ΣPCBs and ΣDDT) in all fish (swordfish, tropical tunas, silky shark and Indian mackerel) from the Seychelles.

Following on from these initial studies, the potential variables of some HNPs were also explored. For instance, occurrence of Q1 (Cl_7_-MBP) was often accompanied with other polyhalogenated 1′-methyl-1,2′-bipyrroles (PMBPs; Cl_x_Br_y_-MBP, x+y=7) (Hauler et al. 2014; Teuten et al. 2006; Vetter et al. 2007). Here, the isomer pattern of BrCl_6_-MBPs and Br_2_Cl_5_-MBPs was the same in Indian mackerel, swordfish and the tropical tunas (Fig. S4a,b). The same BrCl_6_-MBP and Br_2_Cl_5_-MBPs pattern was also found in sardines and chokka squid from South African coast (South Indian Ocean) (Wu et al. 2019, 2020b), indicating that production of Q1 and PMBPs was similar in larger parts of the Indian Ocean. Interestingly, highly brominated PMBPs (Br_3_Cl_4_-MBP and Br_5_Cl_2_-MBP) were detected in low amounts in swordfish but not in other samples (Fig. S4c,d).

Similarly, BC-10 (usually the only or predominant Br_4_Cl_2_-DBP isomer (Hauler et al. 2013; Tittlemier et al. 1999)) was detected in all fish samples but was frequently below LOQ (Table 3). However, similar Br_4_Cl_2_-DBP isomer patterns, namely, predominance of BC-10 with low but distinct contributions of other Br_4_Cl_2_-DBPs could be detected in all fish samples except silky shark and skipjack tuna (low concentrations of BC-10 and other PDBPs, Fig. S5a). Likewise, Br_5_Cl-DBPs (two isomers) were detected in all swordfish samples (except in the liver of F10, Fig. S5b) but not in other fish samples from the Seychelles. Previously, two Br_5_Cl-DBP isomers were also detected in Australian humpback dolphin (*Sousa chinensis*) and sea cucumber (*Holothuria* sp*.*) (Hauler et al. 2013). The widespread occurrence of HNPs in Seychelles prompted us to investigate also tropical tuna samples collected from other regions of the Western Indian Ocean.

### HNPs and POPs in tropical tunas from other regions in the Western Indian Ocean (Chagos, Somalia and Mozambique)

**Fig. 6:**
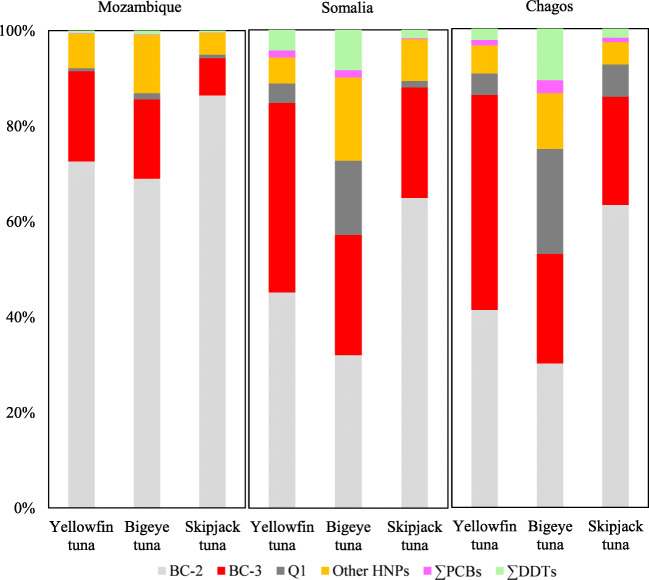
Contributions (%) of different HNPs and anthropogenic POPs to the total amount of polyhalogenated compounds in yellowfin tuna, bigeye tuna, skipjack tuna from the Mozambique, Somalia and Chagos regions in the Western Indian Ocean

In agreement with our findings in the swordfish and tropical tuna species collected from the Seychelles, total contaminant levels in the three tropical tuna muscle samples (one yellowfin tuna, one bigeye tuna and one skipjack tuna, respectively) from Chagos, Somalia and Mozambique (i.e. in the east, northwest and southwest of Seychelles, distance ~2000 km, respectively, Fig. S1) were also dominated by ΣHNPs (Fig. 6). BC-2 (170–3350 ng/g lw) and BC-3 (60–440 ng/g lw), followed by Q1 (6.3-150 ng/g lw), were also the predominant contaminants in all samples from these three regions (Table 4). The individual tropical tuna muscle samples from Mozambique showed remarkably high HNP levels of up to 3860 ng/g lw (with a main contribution of BC-2). This concentration was among the highest ΣHNP levels determined to date in fish (Covaci et al. 2008; Estrella et al. 2018; Kelly et al. 2008). Interestingly, the Mozambique Channel has been described as an important feeding area for tropical tunas (Chassot et al. 2019). Hence, it would be interesting to screen HNPs in further species from this area.

**Table 4:** Concentrations of HNPs and anthropogenic POPs (ng/g lw) in the muscle of three tropical tuna species from Chagos, Somalia and Mozambique in the Western Indian Ocean

	Sampling date	BC-2	BC-3	Q1	BC-11	BC-1	BC-10	Br_6_-DBP	2,4,6-TBA	MHC-1	∑HNPs	∑DDTs	∑PCBs
Chagos													
Yellowfin tuna	Jul.2013	400	440	44	<LOD	19	<LOQ	35	3.0	<LOQ	940	23	11
Bigeye tuna	Jul.2013	200	150	150	20	10	38	<LOQ	1.9	7.0	570	71	18
Skipjack tuna	Jul.2013	170	60	18	<LOD	10	<LOQ	<LOQ	2.4	<LOQ	260	4.9	2.5
Somalia													
Yellowfin tuna	Jun.2013	190	170	17	<LOD	11	<LOQ	10	1.2	0.6	400	18	6.2
Bigeye tuna	Mar.2013	200	160	99	32	12	36	26	1.6	3.1	570	54	9.6
Skipjack tuna	May.2013	1170	420	24	<LOD	58	<LOQ	98	1.8	<LOQ	1770	31	4.4
Mozambique													
Yellowfin tuna	Apr.2013	750	190	6.3	<LOD	18	<LOQ	53	2.1	1.2	1020	5.4	0.7
Bigeye tuna	Mar.2013	1350	330	26	110	33	<LOQ	91	3.4	3.3	1950	15	1.5
Skipjack tuna	Mar.2013	3350	300	28	<LOD	45	<LOQ	128	1.6	3.1	3860	13	3.3

2,4,6-TBP was <LOD in all samples

In addition, PMBP patterns (i.e. BrCl_6_-MBPs and Br_2_Cl_5_-MBPs) and PDBP patterns (Br_4_Cl_2_-DBPs) agreed with those determined in the Seychelles samples, when these HNPs were detectable. Also, concentrations of ΣDDTs (4.9–71 ng/g lw) and ΣPCBs (0.7–18 ng/g lw) in these tropical tuna samples were similarly low as in the samples from the Seychelles (3.5–60 and 1.4–15 ng/g lw) (Table 3).

Based on the typical lipid content of fresh swordfish (12%) and tuna (0.7%) (Sirot et al. 2008), the estimated daily intake (EDI) of ΣPCBs and ΣDDTs via the consumption of swordfish and tropical tuna would pose no risk to human consumers, with concentrations being 3–600 times lower than the threshold, depending on the species and contaminants (Table S4). However, the EDI of ΣHNPs of up to 7080 ng via swordfish and 2700 ng via tropical tuna merits further attention, as the toxicity of HNPs is poorly understood at present.

### Non-target GC/ECNI-MS-SIM analysis of one swordfish sample (F1)

Since determinations via GC/MS-SIM may overlook untargeted polyhalogenated compounds, it appeared meaningful to exemplarily screen the swordfish sample with the highest HNP contamination level (sample F1) by the non-target GC/MS analysis method of Vetter et al. (2017). Altogether, ~80 polyhalogenated compounds were detected in the swordfish sample including the 19 (~25%) compounds covered by the targeted GC/MS-SIM quantification. However, none of the ~60 non-targeted polyhalogenated compounds belonged to the most abundant peaks in the resulting high mass GC/ECNI-MS-SIM chromatograms. As a consequence, most of these compounds could not be identified from the mass spectra produced. However, the following rarely reported HNPs could be tentatively assigned along with traces of the anthropogenic *cis*-chlordane, *trans*-chlordane and *trans*-nonachlor (< 1 ng/g lw).

**Fig. 7:**
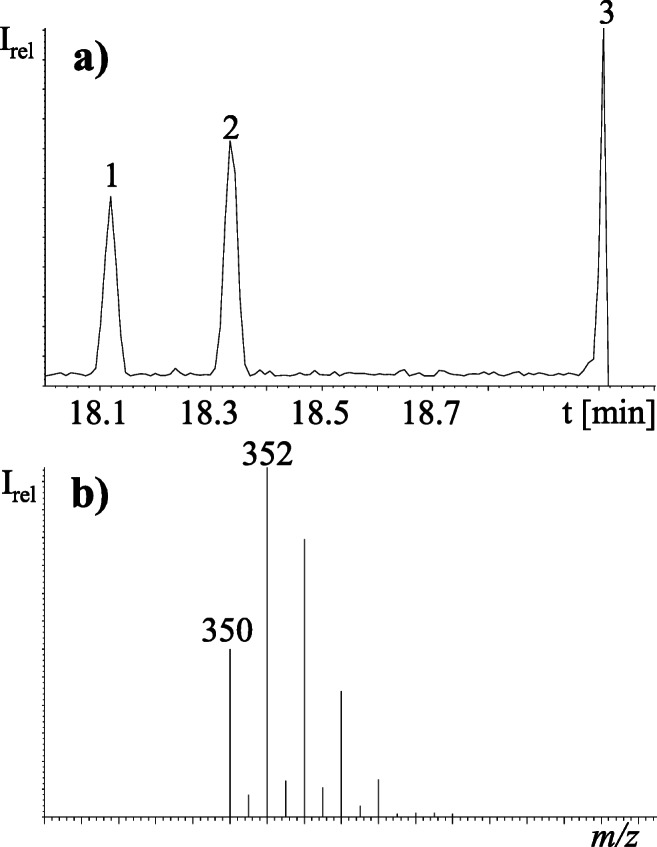
**a** GC/ECNI-MS chromatograms of potential isomers of Cl_6_-MBPs (compounds 1–3) monitored in non-target GC/ECNI-MS-SIM run 1 (NT1) as recorded with *m/z* 352 and **b** their mass spectrum

**Fig. 8:**
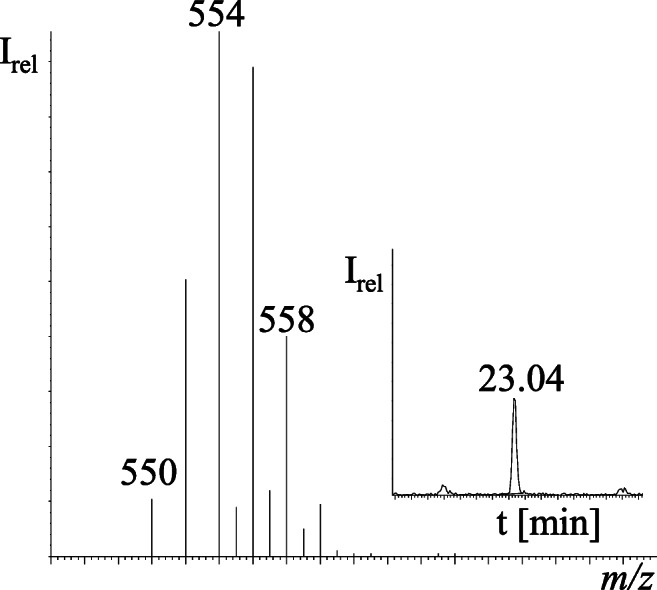
Merged mass spectrum of potential Br_5_-DBPs at the retention time of 23.04 min (non-target GC/ECNI-MS-SIM runs NT1 and NT12)

Three out of five possible Cl_6_-MBPs (one chlorine atom less than Q1) were detected by means of the monoisotopic peak at *m/z* 350 and a very good match of the corresponding hexachlorinated pattern in run NT1 (ratios of *m/z* 350/352/354/356/358 with 51:100:81:35:9 in theory and 48:100:80:36:11 being measured, Fig. 7a, b). Relative retention times (RRTs, relative to Q1 = 1.00) of Cl_6_-MBP isomers **1**, **2** and **3** of 0.91, 0.92 and 0.96, respectively, were similar to those obtained during the UV dehalogenation of Q1 (Cl_6_-MBPs) and subsequently detected in brown skua (*C. skua lonnbergi*) from the Antarctic and melon-headed whale (*Peponocephala electra*) from Australia (Gaul and Vetter 2008), but an unequivocal assignment of structures could not be achieved. Based on the most abundant isotopic peak of the molecular ion of Cl_6_-MBPs (*m/z* 352) and Q1 (*m/z* 386), the estimated abundance of the Cl_6_-MBP isomers **1**, **2** and **3** was 0.1–0.4% of Q1, respectively. Other hydrodehalogenated PMBPs, such as Br_5_Cl-MBP and Br_6_-MBP, were previously detected in the blubber of a common dolphin (*Delphinus delphis*) from Orleans (MA, USA) by means of a non-targeted GC×GC/TOF-MS method (Hoh et al. 2012). In addition, the pentabromo isotope pattern of the compound eluting at 23.04 min was split between NT1 (*m/z* 554–560) and NT12 (*m/z* 550–554). Merging of both halves of the fragmental isotope patterns corresponded with C_10_H_7_Br_5_N_2_ based on the good agreement of measured isotope peak ratios of *m/z* 550/552/554/556/558/560 (11:52:100:93:42:10 measured vs. 11:51:100:98:48:10 in theory) (Fig. 8). Since Br_6_-DBP (C_10_H_6_Br_6_N_2_) was also detected in this swordfish sample, C_10_H_7_Br_5_N_2_ was most likely Br_5_-DBP (one bromine atom less than Br_6_-DBP). Consistent with our results, Br_5_-DBP was previously detected in the blubber of bottlenose dolphins (*Tursiops truncatus*) from the Southern California Bight (Shaul et al. 2015). In both cases, it remained unclear whether Cl_6_-MBPs and Br_5_-DBP are actual HNPs or metabolites of Q1 and Br_6_-DBP. The above-mentioned compounds (Cl_6_-MBPs and Br_5_-DBP) are supposed by the low-resolution GC/ECNI-MS. In subsequent studies, high-resolution MS data with appropriate mass accuracy could be used to verify the molecular formulas.

## Conclusions

Analysis of 24 fish samples from the Seychelles and other regions in the Western Indian Ocean evidenced a pronounced predominance of HNPs over anthropogenic POPs. Namely, HNPs usually contributed to ~70–90% to the total contamination by polyhalogenated compounds. The non-targeted GC/ECNI-MS analysis did not indicate the presence of abundant HNPs in addition to those measured by targeted analysis. However, HNP patterns were less uniform than POP patterns and more dependent on regional (and species-related) differences. Top HNP concentrations in tropical tuna from Mozambique were among the highest reported to date in fish. Especially, the high contamination level with BC-2 and BC-3 was remarkable. The quantity of HNPs ingested by consuming tropical tuna and swordfish were high, and given the widespread occurrence and less predictable distribution, paired with the lack of toxic data, further research should focus on the pollution of large marine predators by HNPs.

## Supplementary information

ESM 1 (PDF 698 kb)

## Data Availability

The authors confirm the data generated or analysed during this study are included in this published article and its supplementary information files.

## References

[CR1] Alonso MB, Maruya KA, Dodder NG, Lailson-Brito J, Azevedo A, Santos-Neto E (2017). Nontargeted screening of halogenated organic compounds in bottlenose dolphins (*Tursiops truncatus*) from Rio de Janeiro. Brazil Environ Sci Technol.

[CR2] Aptula AO, Netzeva TI, Valkova IV, Cronin MTD, Schultz TW, Kühne R et al (2002) Multivariate discrimination between modes of toxic action of phenols. Quant Struct Act Relat 21:12–22. 10.1002/1521-3838(200205)21:1<12::AID-QSAR12>3.0.CO;2-M

[CR3] Barón E, Hauler C, Gallistl C, Giménez J, Gauffier P, Castillo JJ, Fernández-Maldonado C, de Stephanis R, Vetter W, Eljarrat E, Barceló D (2015). Halogenated natural products in dolphins: brain-blubber distribution and comparison with halogenated flame retardants. Environ Sci Technol.

[CR4] Bendig P, Hägele F, Vetter W (2013). Widespread occurrence of polyhalogenated compounds in fat from kitchen hoods. Anal Bioanal Chem.

[CR5] Bidleman TF, Andersson A, Jantunen LM, Kucklick JR, Kylin H, Letcher RJ, Tysklind M, Wong F (2019). A review of halogenated natural products in Arctic. Subarctic and Nordic ecosystems Emerg Contam.

[CR6] Branstetter S (1987). Age, growth and reproductive biology of the silky shark, *Carcharhinus falciformis*, and the scalloped hammerhead, *Sphyrna lewini*, from the northwestern Gulf of Mexico. Environ Biol Fish.

[CR7] Chassot E, Bodin N, Sardenne F, Obura D (2019). The key role of the Northern Mozambique Channel for Indian Ocean tropical tuna fisheries. Rev Fish Biol Fish.

[CR8] Corsolini S, Ademollo N, Romeo T, Greco S, Focardi S (2005). Persistent organic pollutants in edible fish: a human and environmental health problem. Microchem J.

[CR9] Corsolini S, Guerranti C, Perra G, Focardi S (2008). Polybrominated diphenyl ethers, perfluorinated compounds and chlorinated pesticides in swordfish (*Xiphias gladius*) from the Mediterranean Sea. Environ Sci Technol.

[CR10] Covaci A, Losada S, Roosens L, Vetter W, Santos FJ, Neels H, Storelli A, Storelli MM (2008). Anthropogenic and naturally occurring organobrominated compounds in two deep-sea fish species from the Mediterranean Sea. Environ Sci Technol.

[CR11] de Azevedo e Silva CE, Azeredo A, Lailson-Brito J, JPM T, Malm O (2007). Polychlorinated biphenyls and DDT in swordfish (*Xiphias gladius*) and blue shark (*Prionace glauca*) from Brazilian coast. Chemosphere.

[CR12] Estrella LF, Ferreira VB, Gallistl C, Alves MGR, Vetter W, Malm O, Abadio Finco FDB, Torres JPM (2018). Occurrence of halogenated natural products in highly consumed fish from polluted and unpolluted tropical bays in SE Brazil. Environ Pollut.

[CR13] Gaul S, Vetter W (2008). Photolytic dehalogenation of the marine halogenated natural product Q1. Chemospher.

[CR14] Goto A, Tue NM, Isobe T, Takahashi S, Tanabe S, Kunisue T (2020). Nontarget and target screening of organohalogen compounds in mussels and sediment from Hiroshima Bay. Japan: occurrence of novel bioaccumulative substances Environ Sci Technol.

[CR15] Gribble GW (2003) Naturally occurring halogenated pyrroles and Indoles. In: Progress in Heterocyclic Chemistry. Elsevier, pp 58–74. 10.1016/S0959-6380(03)80005-3

[CR16] Gribble GW (2012). Occurrence of halogenated alkaloids. The Alkaloids Chemistry and Biology.

[CR17] Haldén AN, Nyholm JR, Andersson PL, Holbech H, Norrgren L (2010). Oral exposure of adult zebrafish (*Danio rerio*) to 2,4,6-tribromophenol affects reproduction. Aquat Toxicol.

[CR18] Hauler C, Vetter W (2015). A non-targeted gas chromatography/electron capture negative ionization mass spectrometry selected ion monitoring screening method for polyhalogenated compounds in environmental samples. Rapid Commun Mass Sp.

[CR19] Hauler C, Martin R, Knölker H-J, Gaus C, Mueller JF, Vetter W (2013). Discovery and widespread occurrence of polyhalogenated 1,1'-dimethyl-2,2'-bipyrroles (PDBPs) in marine biota. Environ Pollut.

[CR20] Hauler C, Rimkus G, Risacher C, Knölker H-J, Vetter W (2014). Concentrations of halogenated natural products versus PCB 153 in bivalves from the North and Baltic Seas. Sci Total Environ.

[CR21] Hoh E, Dodder NG, Lehotay SJ, Pangallo KC, Reddy CM, Maruya KA (2012). Nontargeted comprehensive two-dimensional gas chromatography/time-of-flight mass spectrometry method and software for inventorying persistent and bioaccumulative contaminants in marine environments. Environ Sci Technol.

[CR22] Hoondert RPJ, van den Brink NW, van den Heuvel-Greve MJ, Ragas AJ, Hendriks AJ (2020). Implications of trophic variability for modeling biomagnification of POPs in marine food webs in the Svalbard archipelago. Environ Sci Technol.

[CR23] Hop H, Borgá K, Gabrielsen GW, Kleivane L, Skaare JU (2002). Food web magnification of persistent organic pollutants in poikilotherms and homeotherms. Environ Sci Technol.

[CR24] Johnson JE, Marshall PA (2007) Climate change and the Great Barrier Reef: a vulnerability assessment. Flinders, Australia: Great Barrier Reef Marine Park Authority

[CR25] Kelly BC, Ikonomou MG, Blair JD, Gobas FAPC (2008). Hydroxylated and methoxylated polybrominated diphenyl ethers in a Canadian Arctic marine food web. Environ Sci Technol.

[CR26] Kim EJ, Park Y-M, Park J-E, Kim J-G (2014). Distributions of new Stockholm Convention POPs in soils across South Korea. Sci. Total Environ.

[CR27] Letcher RJ, Klasson-Wehler E, Bergman A, Hutzinger O, Paasivirta J (2000). Methyl sulfone and hydroxylated metabolites of polychlorinated biphenyls. Anthropogenic Compounds Part K.

[CR28] Löfstrand K, Malmvärn A, Haglund P, Bignert A, Bergman A, Asplund L (2010). Brominated phenols, anisoles, and dioxins present in blue mussels from the Swedish coastline. Environ Sci Pollut R.

[CR29] Malmvärn A, Marsh G, Kautsky L, Athanasiadou M, Bergman A, Asplund L (2005). Hydroxylated and methoxylated brominated diphenyl ethers in the red algae *Ceramium tenuicorne* and blue mussels from the Baltic Sea. Environ Sci Technol.

[CR30] Marsili L, Jiménez B, Borrell A (2018) Persistent organic pollutants in cetaceans living in a hotspot area: the Mediterranean Sea. In: Marine Mammal Ecotoxicology. Elsevier, pp 185–212. 10.1016/B978-0-12-812144-3.00007-3

[CR31] Munschy C, Vigneau E, Bely N, Héas-Moisan K, Olivier N, Pollono C, Hollanda S, Bodin N (2020). Legacy and emerging organic contaminants: levels and profiles in top predator fish from the western Indian Ocean in relation to their trophic ecology. Environ Res.

[CR32] Nyholm JR, Norman A, Norrgren L, Haglund P, Andersson PL (2009). Uptake and biotransformation of structurally diverse brominated flame retardants in zebrafish (*Danio rerio*) after dietary exposure. Environ Toxicol Chem.

[CR33] Qiu YW, Wang DX, Zhang G (2020). Assessment of persistent organic pollutants (POPs) in sediments of the Eastern Indian Ocean. Sci Total Environ.

[CR34] Rosenfelder N, Vetter W (2012). Stable carbon isotope composition (δ^13^C values) of the halogenated monoterpene MHC-1 as found in fish and seaweed from different marine regions. J Environ Monit.

[CR35] Sardenne F, Bodin N, Chassot E, Amiel A, Fouché E, Degroote M, Hollanda S, Pethybridge H, Lebreton B, Guillou G, Ménard F (2016). Trophic niches of sympatric tropical tuna in the Western Indian Ocean inferred by stable isotopes and neutral fatty acids. Prog Oceanogr.

[CR36] Shaul NJ, Dodder NG, Aluwihare LI, Mackintosh SA, Maruya KA, Chivers SJ, Danil K, Weller DW, Hoh E (2015). Nontargeted biomonitoring of halogenated organic compounds in two ecotypes of bottlenose dolphins (*Tursiops truncatus*) from the Southern California Bight. Environ Sci Technol.

[CR37] Sirot V, Oseredczuk M, Bemrah-Aouachria N, Volatier J-L, Leblanc J-C (2008). Lipid and fatty acid composition of fish and seafood consumed in France: CALIPSO study. J Food Compos Anal.

[CR38] Sonne C, Siebert U, Gonnsen K, Desforges J-P, Eulaers I, Persson S, Roos A, Bäcklin BM, Kauhala K, Tange Olsen M, Harding KC, Treu G, Galatius A, Andersen-Ranberg E, Gross S, Lakemeyer J, Lehnert K, Lam SS, Peng W, Dietz R (2020). Health effects from contaminant exposure in Baltic Sea birds and marine mammals: a review. Environ Int.

[CR39] Stefanelli P, Ausili A, Di Muccio A, Fossi C, Di Muccio S, Rossi S (2004). Organochlorine compounds in tissues of swordfish (*Xiphias gladius*) from Mediterranean Sea and Azores Islands. Mar Pollut Bull.

[CR40] Teuten EL, Reddy CM (2007). Halogenated organic compounds in archived whale oil: a pre-industrial record. Environ Pollut.

[CR41] Teuten EL, Xu L, Reddy CM (2005). Two abundant bioaccumulated halogenated compounds are natural products. Science.

[CR42] Teuten EL, Pedler BE, Hangsterfer AN, Reddy CM (2006). Identification of highly brominated analogues of Q1 in marine mammals. Environ Pollut.

[CR43] Tittlemier SA, Simon M, Jarman WM, Elliott JE, Norstrom RJ (1999). Identification of a novel C_10_H_6_N_2_Br_4_Cl_2_ heterocyclic compound in seabird eggs. A bioaccumulating marine natural product?. Environ Sci Technol.

[CR44] Vetter W (2006). Marine halogenated natural products of environmental relevance. Rev Environ Contam T.

[CR45] Vetter W (2012) Polyhalogenated alkaloids in environmental and food samples. In: The Alkaloids. Chemistry and Biology, Elsevier, 71:211–276. 10.1016/B978-0-12-398282-7.00003-5 10.1016/b978-0-12-398282-7.00003-5 23189748

[CR46] Vetter W, Scholz E, Gaus C, Müller JF, Haynes D (2001). Anthropogenic and natural organohalogen compounds in blubber of dolphins and dugongs (*Dugong dugon*) from northeastern Australia. Arch Environ Contam Toxicol.

[CR47] Vetter W, Stoll E, Garson MJ, Fahey SJ, Gaus C, Müller JF (2002). Sponge halogenated natural products found at parts-per-million levels in marine mammals. Environ Toxicol Chem.

[CR48] Vetter W, Gaul S, Olbrich D, Gaus C (2007). Monobromo and higher brominated congeners of the marine halogenated natural product 2,3,3',4,4',5,5'-heptachloro-1'-methyl-1,2'-bipyrrole (Q1). Chemospher.

[CR49] Vetter W, Haase-Aschoff P, Rosenfelder N, Komarova T, Mueller JF (2009). Determination of halogenated natural products in passive samplers deployed along the Great Barrier Reef. Queensland/Australia Environ Sci Technol.

[CR50] Vetter W, Gallistl C, Schlienz A, Preston T, Müller J, von der Trenck KT (2017). Brominated flame retardants (BFRs) in eggs from birds of prey from Southern Germany, 2014. Environ Pollut.

[CR51] von der Recke R, Vetter W (2007). Synthesis and characterization of 2,3-dibromopropyl-2,4,6-tribromophenyl ether (DPTE) and structurally related compounds evidenced in seal blubber and brain. Environ Sci Technol.

[CR52] Voorspoels S, Covaci A, Schepens P (2003). Polybrominated diphenyl ethers in marine species from the Belgian North Sea and the Western Scheldt Estuary: levels, profiles, and distribution. Environ Sci Technol.

[CR53] Weijs L, Losada S, Das K, Roosens L, Reijnders PJH, Santos JF, Neels H, Blust R, Covaci A (2009). Biomagnification of naturally-produced methoxylated polybrominated diphenyl ethers (MeO-PBDEs) in harbour seals and harbour porpoises from the southern North Sea. Environ Int.

[CR54] Wenning RJ, Martello L (2014) POPs in marine and freshwater environments. In: Environmental Forensics for Persistent Organic Pollutants, Elsevier, pp 357–390. 10.1016/B978-0-444-59424-2.00008-6

[CR55] Wilson S (2018). AMAP Assessment 2016: Chemicals of Emerging Arctic Concern.

[CR56] Wu Q, Bouwman H, Uren RC, van der Lingen CD, Vetter W (2019). Halogenated natural products and anthropogenic persistent organic pollutants in chokka squid (*Loligo reynaudii*) from three sites along the South Atlantic and Indian Ocean coasts of South Africa. Environ Pollut.

[CR57] Wu Q, Krauß S, Vetter W (2020). Occurrence and fate studies (sunlight exposure and stable carbon isotope analysis) of the halogenated natural product MHC-1 and its producer *Plocamium cartilagineum*. Sci Total Environ.

[CR58] Wu Q, Schlag S, Uren R, van der Lingen CD, Bouwman H, Vetter W (2020). Polyhalogenated compounds (halogenated natural products and POPs) in sardine (*Sardinops sagax*) from the South Atlantic and Indian Oceans. J Agric Food Chem.

