# Supplementary material for: High levels of halogenated natural products in large pelagic fish from the Western Indian Ocean
Source: Environ Sci Pollut Res Int. 2021 Jun 15;28(39):55252–64. doi: 10.1007/s11356-021-14738-0 (PMC8494675; doi:10.1007/s11356-021-14738-0)
Supplement: Supplementary file 1 — (PDF 698 kb) [file 11356_2021_14738_MOESM1_ESM.pdf]

Supplementary materials for:

High levels of halogenated natural products in large pelagic fish from the Western Indian Ocean

Qiong Wu[1], Catherine Munschy[2], Yann Aminot[2], Nathalie Bodin[3,4†], Walter Vetter[1]*

[1]University of Hohenheim, Institute of Food Chemistry (170b), Garbenstraße 28, D-70599 Stuttgart, Germany

[2] IFREMER (French Research Institute for Exploitation of the Sea), Laboratory of Biogeochemistry of Organic Contaminants, Rue de l'île d'Yeu, BP 21105, 44311 Nantes Cedex 3, France

[3] SFA (Seychelles Fishing Authority), Fishing Port, Victoria, Mahé, Seychelles

[4] IRD (French Research Institute for Sustainable Development)

Fishing Port, Victoria, Mahé, Seychelles

[†] present address: SOS (Sustainable Ocean Seychelles), BeauBelle, Mahé, Seychelles

*Corresponding author

Tel: +49 711 459 24016

Fax: +49 711 459 24377

E-mail: walter.vetter@uni-hohenheim.de

Table S1 Details on the fish species investigated in the present study. Length (cm) corresponds to the low jaw fork length for the swordfish, the fork length for the three tropical tunas and the Indian mackerel, and the standard length for the silky shark. Lipids corresponds to the total lipid content estimated on the freeze-dried muscle, and expressed in percent of dry weight (% dw)

Species	Region	Date	n	Gender*	Length	Lipids	Diet	Hunting range
Swordfish	Seychelles	Jan.2013-Nov.2014	10	5x F, 5x M	121-204	17-61	Carnivore (fishes, crustaceans, cephalopods etc.)	Opportunist, feeds deeper
Yellowfin tuna	Seychelles	Feb.2013	1	M	142	1.3	Carnivore (fishes, crustaceans, cephalopods etc.)	Surface and subsurface
Bigeye tuna	Seychelles	Apr.2013	1	M	91	1.3	Carnivore (fishes, crustaceans, cephalopods etc.)	Feeds deeper than other tunas
Skipjack tuna	Seychelles	Apr.2013	1	F	55	0.9	Carnivore (fishes, crustaceans, cephalopods etc.)	Surface and subsurface
Silky shark	Seychelles	Aug.2018	1	M	69 (new born)	2.7	Carnivore (fishes, crustaceans, cephalopods etc.)	From the surface to a depth of 200 m
Indian mackerel	Seychelles	Mar.2018	1	M	26	1.6	Carnivore (macroplankton such as larval shrimps, fish)	Planktivore
Yellowfin tuna	Chagos	Jul.2013	1	M	156	1.2	Carnivore (fishes, crustaceans, cephalopods etc.)	Surface and subsurface
Bigeye tuna	Chagos	Jul.2013	1	M	149	1.0	Carnivore (fishes, crustaceans, cephalopods etc.)	Feeds deeper than other tunas
Skipjack tuna	Chagos	Jul.2013	1	M	72	0.9	Carnivore (fishes, crustaceans, cephalopods etc.)	Surface and subsurface
Yellowfin tuna	Somalia	Jul.2013	1	M	152	3.7	Carnivore (fishes, crustaceans, cephalopods etc.)	Surface and subsurface
Bigeye tuna	Somalia	Mar.2013	1	M	135	1.8	Carnivore (fishes, crustaceans, cephalopods etc.)	Feeds deeper than other tunas
Skipjack tuna	Somalia	May.2013	1	M	66	0.8	Carnivore (fishes, crustaceans, cephalopods etc.)	Surface and subsurface
Yellowfin tuna	Mozambique	Apr.2013	1	M	108	3.3	Carnivore (fishes, crustaceans, cephalopods etc.)	Surface and subsurface
Bigeye tuna	Mozambique	Mar.2013	1	I	54	1.6	Carnivore (fishes, crustaceans, cephalopods etc.)	Feeds deeper than other tunas
Skipjack tuna	Mozambique	Mar.2013	1	M	58	1.0	Carnivore (fishes, crustaceans, cephalopods etc.)	Surface and subsurface

* F = female, M = male, I = indeterminate (juvenile)

Table S2 Limit of detection (LOD) and limit of quantification (LOQ) for polyhalogenated compounds analyzed in present study.

#	Compounds	LOD (pg/µl)	LOQ (pg/µl)
1	2,4,6-TBA	0.02	0.07
2	MHC-1	0.22	0.72
3	BC-2	0.15	0.49
4	BC-1	0.11	0.36
5	BC-10	0.15	0.49
6	BC-3	0.15	0.51
7	BC-11	0.26	0.86
8	Q1	0.01	0.03
9	Br_6-DBP	1.28	4.25
10	PCB 101	0.05	0.15
11	PCB 118	0.01	0.02
12	PCB 153	0.01	0.02
13	PCB 138	0.01	0.03
14	PCB 180	0.01	0.03

Table S3 Mean concentrations (ng/g lw) of individual PCB and DDT congeners in the muscle of fish collected from Seychelles waters in the Western Indian Ocean.

	PCB 101	PCB 118	PCB 138	PCB 153	PCB 180	*o,p'*-DDE	*p,p'*-DDE	*p,p'*-DDD	*o,p'*-DDT	*p,p'*-DDT
Swordfish	0.5	0.5	2.0	2.1	1.6	0.3	26	0.2	15	18
Yellowfin tuna	<LOQ	0.3	1.7	1.9	2.1	<LOQ	13	<LOQ	<LOQ	0.3
Bigeye tuna	0.5	0.2	0.4	0.5	0.4	<LOQ	5.8	<LOQ	<LOQ	<LOQ
Skipjack tuna	5.8	2.7	3.2	2.6	0.6	<LOQ	1.8	<LOQ	<LOQ	13
Silky shark	<LOQ	0.1	0.3	0.7	0.4	<LOQ	6.0	<LOQ	<LOQ	<LOQ
Indian mackerel	<LOQ	<LOQ	0.3	0.5	0.6	<LOQ	3.5	<LOQ	<LOQ	<LOQ

*PCB 28 and PCB 52 were not detected in any samples; LOQ = limit of quantification.

Table S4 Estimated daily intake (EDI) (ng) of ΣHNPs, ΣDDTs and ΣPCBs from 100 g fresh fillets and corresponding Reference Dose for Oral Exposure (RfD) (ng).

Compound	EDI (swordfish) [a]	EDI (tuna) [a]	RfD [b]
ΣHNPs	7080	2700	unavailable
ΣDDT	2300	50	30000 [c]
ΣPCBs	370	13	1200 [c]

[a] The highest concentrations of the corresponding compounds in all samples were used to calculate the EDI of swordfish and tuna.

[b] RfD values were obtained from the United States Environmental Protection Agency (U.S.EPA), IRIS (the Integrated Risk Information System); the body weight was defined as 60 kg;

[c] RfD values of *p,p'*-DDT and Aroclor 1254 were used to estimate the RfD values of ΣDDT and ΣPCBs, respectively.

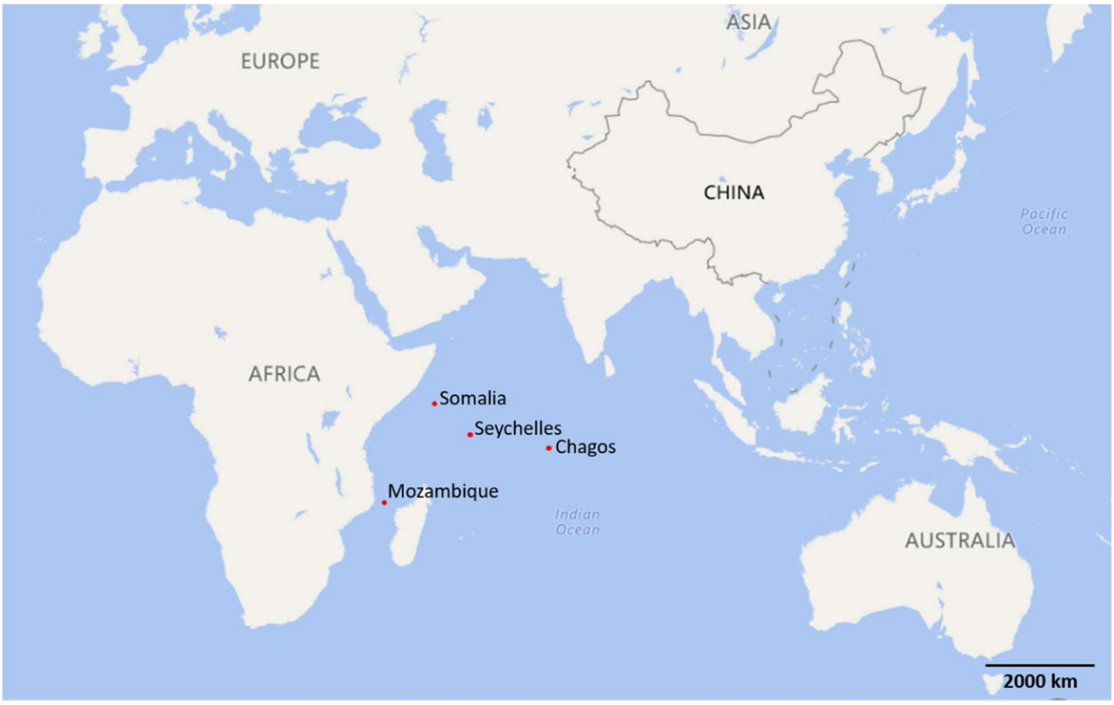

Fig. S1 Sampling map.

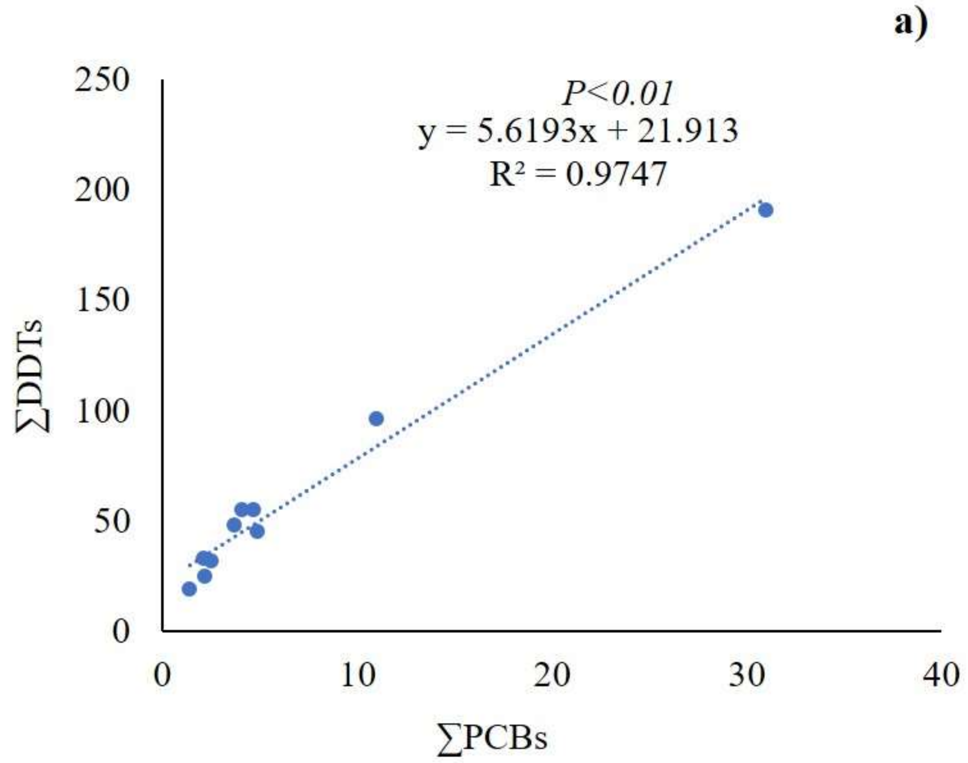

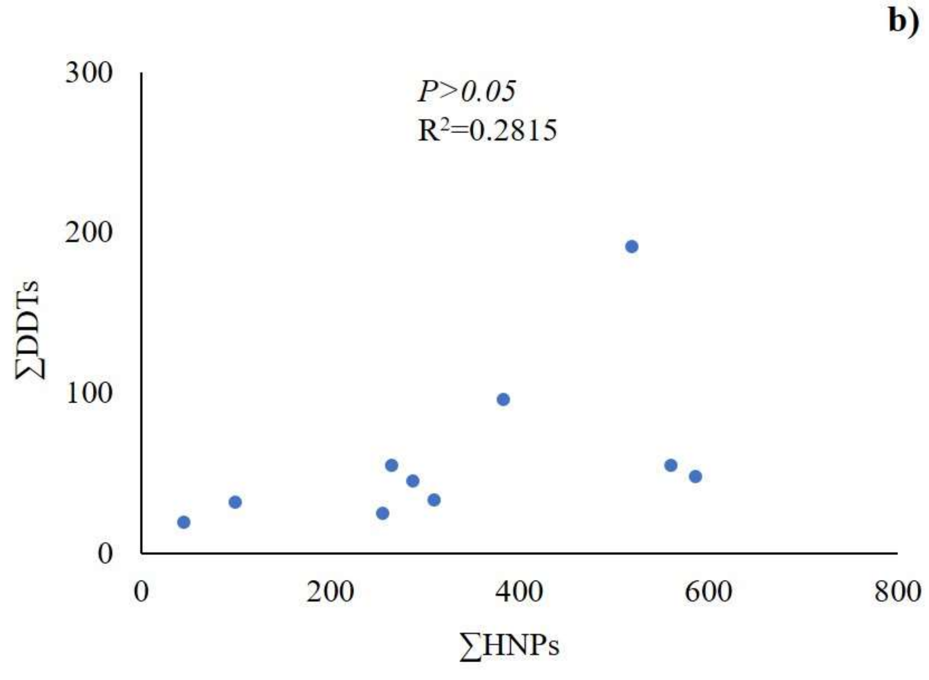

Fig. S2 Correlations between **(a)** ∑PCBs and ∑DDTs as well as **(b)** ∑HNPs and ∑DDTs for ten muscle samples of swordfish from the Seychelles coast. Concentrations are expressed in ng/g lw.

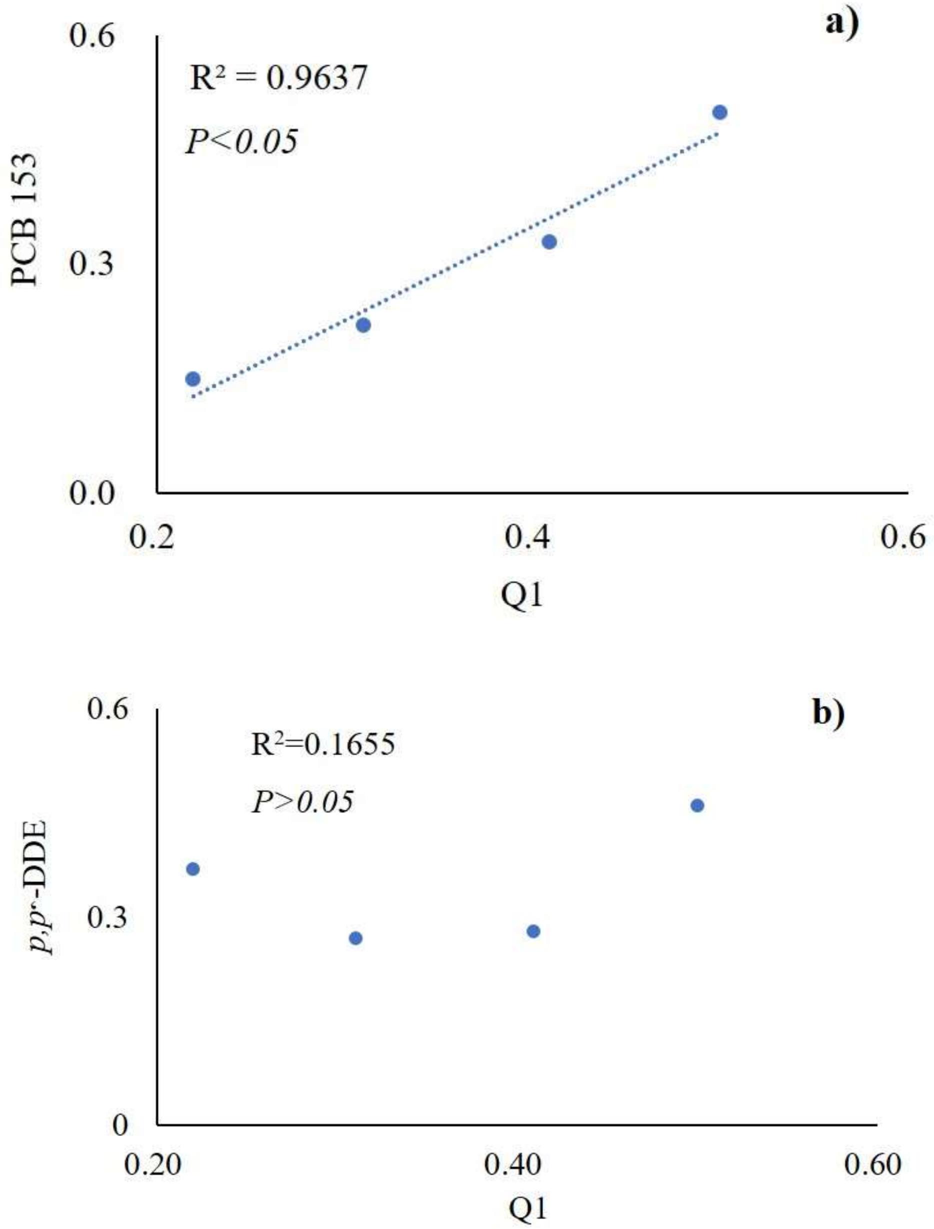

Fig. S3 Correlations between $R_{L/L+F}$ of **(a)** Q1 and PCB 153 as well as **(b)** Q1 and *p,p´*-DDE.

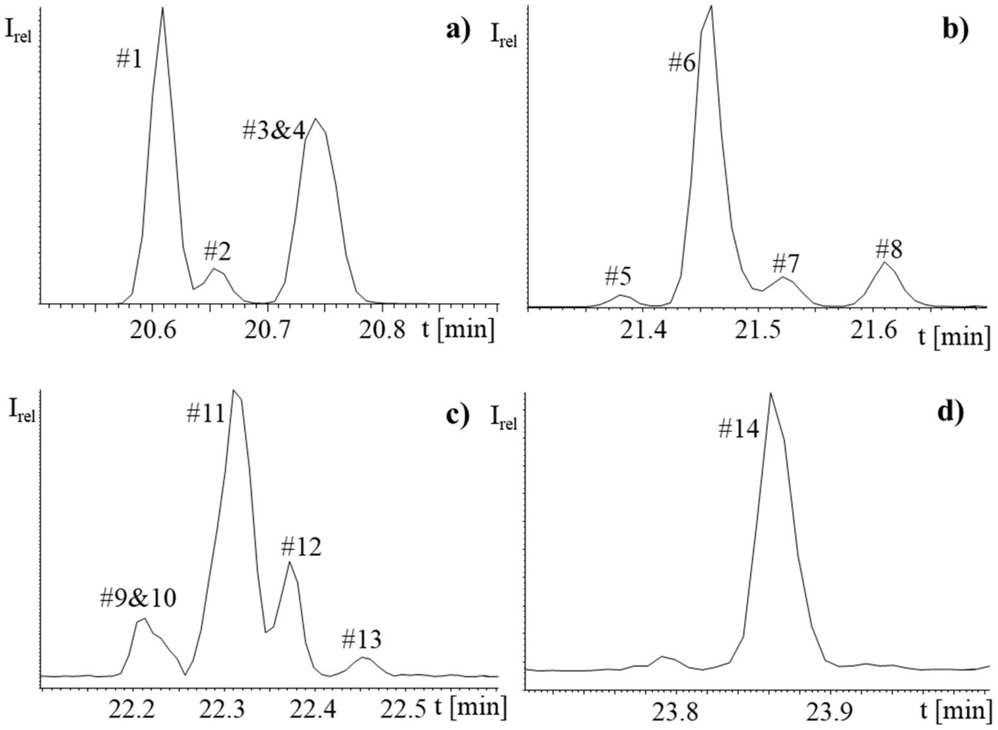

Fig. S4 GC/ECNI-MS chromatograms of **(a)** $BrCl_6$-MBPs (*m/z* 432), **(b)** Br_2Cl_5-MBPs (*m/z* 476), **(c)** Br_3Cl_4-MBPs (*m/z* 520) and **(d)** Br_5Cl_2-MBPs (*m/z* 610) in the present samples.

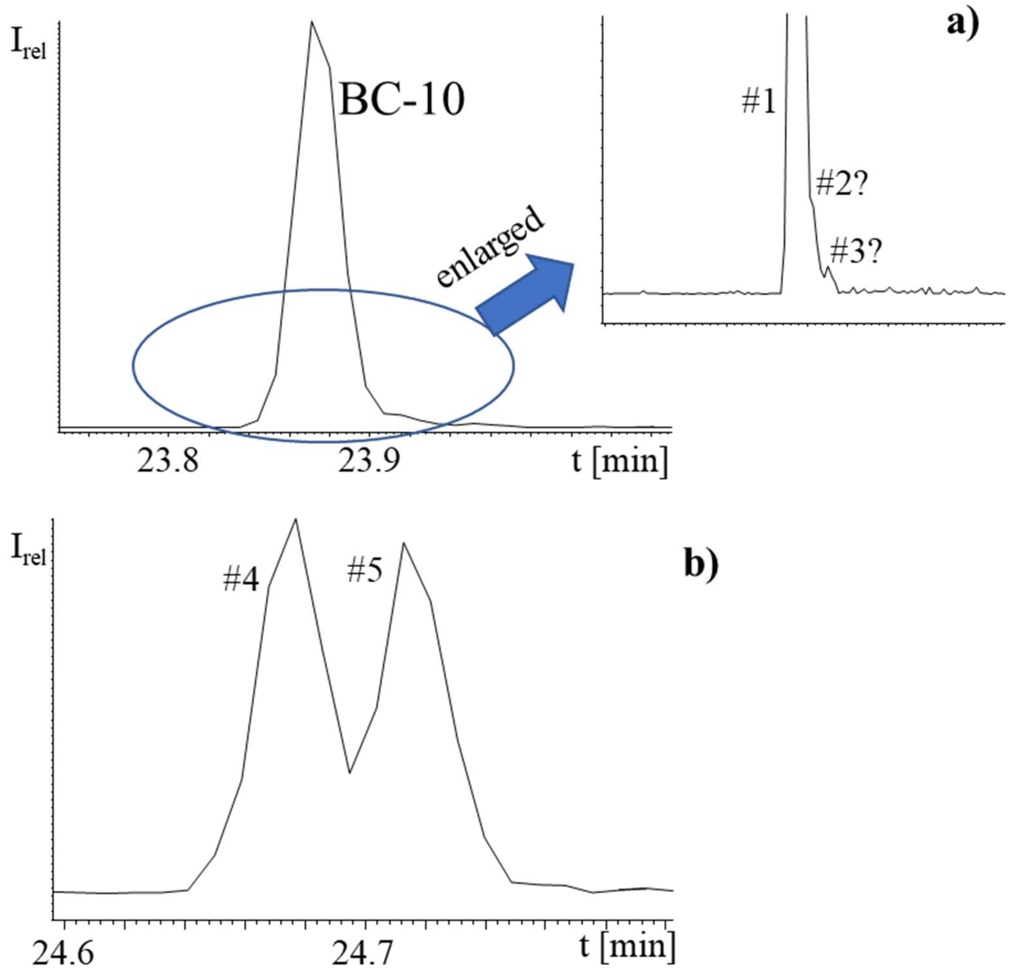

Fig. S5 GC/ECNI-MS chromatograms of **(a)** Br_4Cl_2-DBP (*m/z* 544) and **(b)** Br_5Cl-DBP (*m/z* 590) in the present samples.
